# Glaucocalyxin A suppresses multiple myeloma progression in vitro and in vivo through inhibiting the activation of STAT3 signaling pathway

**DOI:** 10.1186/s12935-021-02375-z

**Published:** 2021-12-19

**Authors:** Mei Li, Cailong Chen, Qian Wang, Xiaolu Jiang, Lanlan Tan, Ying Huang, Yan Zhang, Zubin Zhang

**Affiliations:** 1 Institute of Pediatric Research, Children’s Hospital of Soochow University, Suzhou, 215025 China; 2 Children Health Management Center, Children’s Hospital of Soochow University, Suzhou, 215025 China; 3 Department of Anesthesiology, Children’s Hospital of Soochow University, Suzhou, 215025 China; 4 Department of Ultrasound, The First Affiliated Hospital of Soochow University, Suzhou, 215123 China; 5 Department of Gynecology and Obstetrics, Wuxi Maternal and Child Health Hospital, The Affiliated Hospital of Nanjing Medical University, Wuxi, 214002 Jiangsu Province China; 6 Jiangsu Key Laboratory of Neuropsychiatric Diseases, Department of Pharmacology, College of Pharmaceutical Sciences, Soochow University, Suzhou, 215123 China

**Keywords:** STAT3, Apoptosis, Cell cycle, Glaucocalyxin A, Multiple myeloma

## Abstract

**Background:**

Multiple myeloma (MM) is the most common malignant hematological disease in the people worldwide. Glaucocalyxin A (GLA) is a bioactive ent-kauranoid diterpenoid, that is derived from *Rabdosia japonica var*. GLA has been demonstrated that it had various pharmacological activities, such as anti-coagulation, anti-bacterial, anti-tumor, anti-inflammation, antioxidant activities. Although GLA has effective anti-tumor properties, its effects on multiple myeloma remain unclear. The aim of this study was to examine the possible anti-cancer effects of GLA and their molecular mechanisms on MM cells in vitro and in vivo.

**Methods:**

To evaluate the role of GLA on the proliferation of MM cells in vitro and in vivo, we used MTT method to detect the role of GLA on the proliferation of MM cells. Cell apoptosis and cell cycle assay were evaluated by flow cytometry. Protein expressions in GLA-treated and untreated MM cells were evaluated by western blot analyses. MM xenograft nude mice model was used to investigate the role of GLA on the proliferation of MM cells in vivo. IHC assay was used to examine the role of GLA on the MM xenograft model in vivo.

**Results:**

In the present study, we firstly reported the potent anti-myeloma activity of GLA on MM cells. We found that GLA could induce apoptosis in vitro and in vivo. GLA could inhibit the phosphorylation of the signal transducer and activator of transcription 3 (STAT3) and downregulate interleukin IL-6 induced STAT3 phosphorylation in MM. Overexpression of STAT3 could significantly prevent apoptosis induced by GLA; while knockdown of STAT3 enhanced it. Moreover, GLA could inhibit cell proliferation by inducing the cell cycle arrest. GLA reduced the expression of cell cycle-related proteins CCNB1, CCND1, CCND2, and CCND3 and increased the expression of p21 in MM cell lines. In addition, in the MM xenograft nude mice model, GLA exhibited very good anti-myeloma activity. Administration of GLA almost completely inhibited tumor growth within 19 days without physical toxicity. And the IHC results showed GLA significantly inhibited cell proliferation and interfered STAT3 pathway on MM xenograft model tumor tissues.

**Conclusions:**

Taken together, our present research indicated that GLA inhibits the MM cell proliferation, induces MM cell apoptosis and cell cycle arrest through blocking the activation of STAT3 pathway. Thus, GLA may be a potential therapeutic candidate for MM patients in the future.

## Background

Multiple myeloma (MM) is a clonal B-cell malignancy, which characterization is the proliferation of malignant plasma cells in the bone marrow, and MM is also the second most common hematological malignancy [1, 2]. There are many drugs that have been used for the treatment of MM, such as thalidomide, lenalidomide, bortezomib, and many small molecular inhibitors [1, 3]. However, due to the limitations of these in the clinical, i.e., drug resistance, and severe side effects, the therapies often failed [2]. Thus, new drugs or new therapeutic mechanisms of novel drugs need to be found to reduce clinical drug limitations.

In recent researches about MM therapies, STAT3 was found highly expressed in many CD138 positive cells isolated from primary MM patients, and its expression is often associated with MM pathogenesis and its chemo-resistance to most of the clinical therapies [4, 5]. Signal transducer and activator of transcription 3 (STAT3) is an oncogenic transcription factor. And it has been demonstrated that it is widely overexpressed in many cancer tissues, including cervical carcinoma, hematological malignancies, breast cancer, gastric cancer, glioma, hepatocellular cancer [6, 7]. After stimulation, phosphorylated STAT3 forms homo- or hetero-dimers, these dimers translocate to the nuclei, binds to DNA and regulates its down-stream gene transcriptional expression, including apoptosis related factors (such as *Mcl-1, Bcl-2),* cell cycle regulators (such as *D-cyclins, E2F-1*), VEGF, NF-κB pathway and so on [5, 6, 8]. Many new drugs recently have been discovered through targeting the STAT3 signaling pathway, such as AZD9150, OPB-31121, and Bosutinib, and they also showed significant inhibition efficiency of many cancers, including multiple myeloma [6, 9].

Glaucocalyxin A is an ent-kauranoid diterpene derived from *Rabdosia japonica var.* It has been demonstrated to have numerous biological activities including inhibition of tumor growth, platelet aggregation, cytotoxic, and anti-tumor activities [10–12]. The main molecular mechanisms in cancers inhibited by Glaucocalyxin A are: Glaucocalyxin A induces the cancer cells apoptosis including leukemia, breast cancers, and glioma [13–15]. Glaucocalyxin A inhibits the cell cycle progression such as bladder cancer cells [16]. However, the anticancer effect of Glaucocalyxin A on multiple myeloma has not been reported till now and the potential mechanisms remain unclear.

In the present study we found Glaucocalyxin A has very good anti-cancer activities on human multiple myeloma. We demonstrated that Glaucocalyxin A induces the cell apoptosis and cell cycle arrest by inhibiting STAT3 signaling pathway in vitro and in vivo. Our findings indicated that Glaucocalyxin A might be an effective candidate for human multiple myeloma in the future.

## Materials and methods

### Reagents

**Fig. 1:**
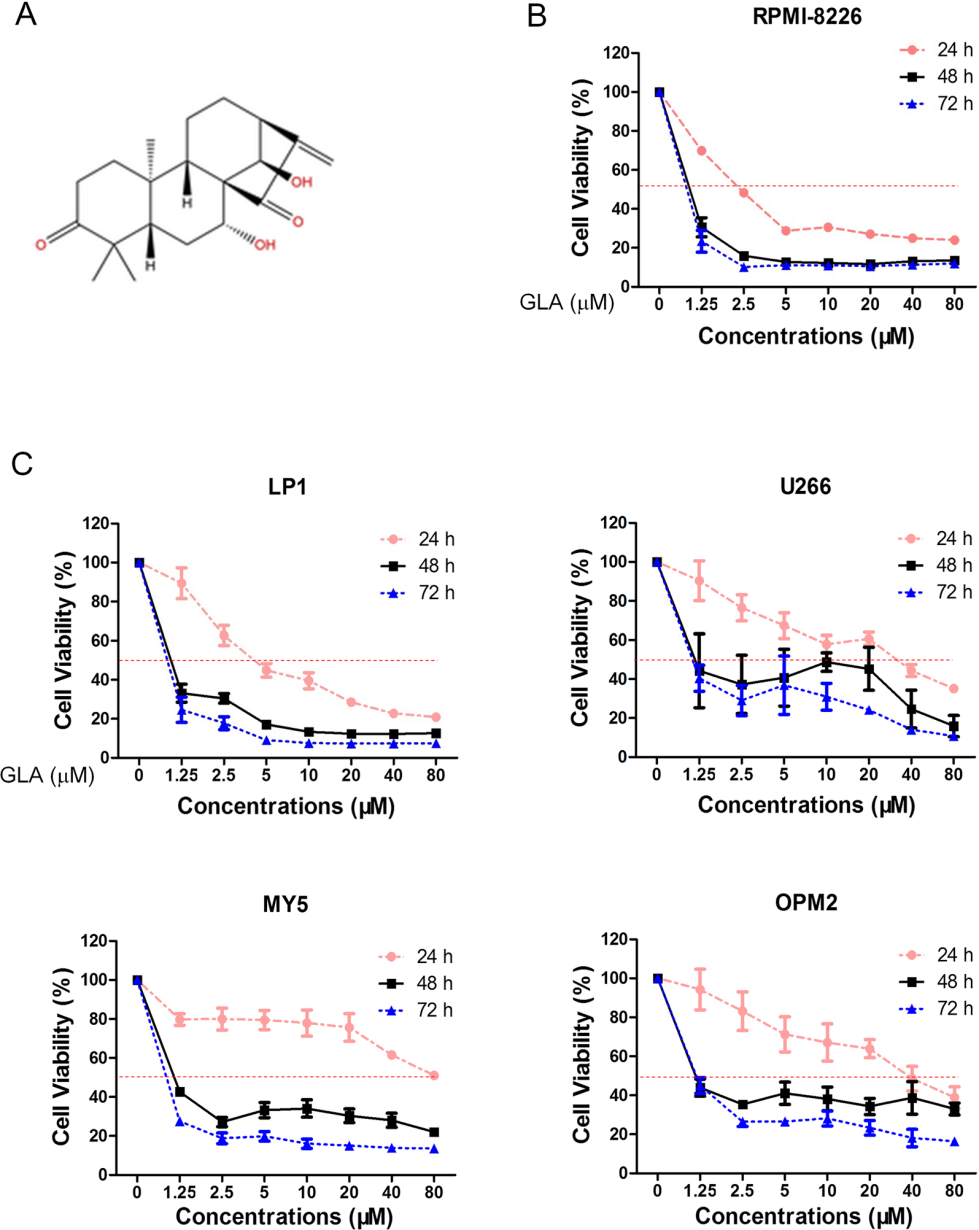
GLA inhibits the growth of MM cell lines. **A** The chemical structure of Glaucocalyxin A (GLA). **B**, **C** The five MM cell lines (RPMI-8226, LP1, U266, OCI-MY5 and OPM2) were treated with the indicated concentrations of GLA for 24, 48 and 72 h, respectively. MTT assays were used to detect the cell viability. The results are shown as means ± SD from three independent experiments

Glaucocalyxin A (purity > 98%) was purchased from Shanghai Yuan Ye Biotechnology Co. Ltd. (Shanghai, China). The Fig. 1A showed its molecular structure (Molecular Formula: C_20_H_28_O_4_; Molecular Weight: 332.437 Da). Glaucocalyxin A was dissolved in dimethyl sulfoxide (DMSO, Sigma, St Louis, USA) and stored at − 80 °C (40 mM stock solution). The cell culture reagents were purchased from Invitrogen Life Technologies (Carlsbad, CA, USA). p-STAT3 (Tyr705) (#9145), STAT3 (#9139), Bcl-2 (#15071), PARP (#9532), CCND1 (#55506), CCND2 (#3741), CCND3 (#2936), CCNB1 (#12231), Caspase 3 (#9662), and cleaved Caspase 3 (#9661) antibodies were purchased from Cell Signaling Technology Inc. (Danvers, MA, USA). The antibodies dilution ratio is 1:1000. Antibodies against anti-mouse immunoglobulin G (IgG) and anti-rabbit IgG horseradish peroxidase conjugated antibody were purchased from Beyotime (Jiangsu, China). Anti-Myc antibody and GAPDH was purchased from Sigma (St. Louis, USA). These two antibodies dilution ratio is 1:10,000. MTT (3-(4,5-dimethylthiazol-2-yl)-2,5-diphenyltetrazolium bromide) was obtained from sigma (St. Louis, USA). Annexin V-FITC and PI detection kit were purchased from Lianke (Zhejiang, China).

### MM cell lines and cell culture

Human MM cell lines U266 and RPMI-8226 were purchased from the American Type Culture Collection (ATCC, Manassas, USA). LP1, OPM2 and OCI-MY5 was kindly given from Professor Xinliang Mao (Soochow University). MM cell lines were cultured in IMDM medium containing 10% fetal bovine serum (FBS, Biological Industries), 100 IU/ml penicillin (Beyotime, China), and 100 μg/ml streptomycin (Beyotime, China). Cells were maintained at 37 °C in 5% CO_2_.

### Cell proliferation assay

MM cells (OPM2, RPMI-8226, U266, LP1 and OCI-MY5) were seeded at a density of 1 × 10^4^ cells per well in 96 cell plate, and the cells were treated with different concentrations of Glaucocalyxin A (0, 1.25, 2.5, 5, 10, 20, 40, 80 μM) for different time (24, 48, and 72 h) as previously described [17]. Before detection, 10 μl of MTT was added to each well and incubated at 37 °C for 4 h. Cells were then dissolved in MTT buffer for 12 h and then were measured at 450 nm to assess proliferation. The cell viability of the MM cells in response to treatment were calculated. Cell viability (%) = (Treated group OD value/Control group OD value) × 100%.

### Cell apoptosis assay

The apoptosis was detected using Annexin V-FITC apoptosis detection kit, the protocol was same as previously described [18]. MM cells (2 × 10^6^) were treated with Glaucocalyxin A and analyzed after 24 h through flow cytometry (Becton Dickinson). The apoptosis rate was detected by flow cytometry.

### Cell-cycle assay

Glaucocalyxin A-treated and -untreated MM cells were collected, washed with iced phosphate buffered saline (PBS) one time. Cells were fixed with 70% cold ethanol at − 20 °C for 12 h, and then cells were centrifuged at 800*g* for 5 min and washed with cold PBS. Finally, cells were stained with PI for 15 min at room temperature in the dark. Then the stained cells were detected by flow cytometry.

### Cell transfection

STAT3 siRNA was purchased from Genepharm Co. Ltd, Suzhou, China. Before transfection, HEK293T and RPMI-8226 cells were seeded at a density of 5 × 10^5^ cells per well in six-well plates. And the STAT3 siRNA (final concentration is 100 nM) were transfected into these cells using Lipofectamine RNAi Max (Invitrogen, USA) as described previously [19].

### Real time *qPCR* assay

As previously described [20], total RNA was extracted using TRIzol (Invitrogen, USA). Reverse transcription reaction was performed using the First-Strand cDNA synthesis kit (TIANGEN, China) and PCR analysis was run using SYBR Green (TIANGEN, China). Reactions were processed and analyzed by Real-Time qPCR System (Bio-Rad, USA). The relative expression level of genes was calculated by the ΔΔCt method. The qPCR primers were synthesized by Sangon tech (Shanghai, China). And the PCR primers are: Bcl-2 F: GCGGATTGACATTTCTGTG, R: CATAAGGCAACGATCCCA; Caspase 3 F: CAGTGATGCTGTGCTATGAAT, R: CAGATGCCTAAGTTCTTCCAC; GAPDH F: CCTTCCGTGTCCCCACT, R: GCCTGCTTCACCACCTTC.

### Western blot

Cells were collected and lysed in lysis buffer (100 mM Tris–HCl, pH 6.8, 4% sodium dodecyl sulfate (SDS), 20% glycerol, and protease inhibitors) at 4 °C for 20 min. Proteins (30 μg) were fractionated by SDS–polyacrylamide gel electrophoresis (PAGE) and transferred onto PVDF membrane. The membranes were blocked with 5% milk at room temperature for 1 h and incubated with primary antibodies overnight at 4 °C, followed by treatment with secondary antibodies at room temperature for 1 h. GADPH was used to normalize the amount of protein in each sample. Blots were then developed in ECL Prime solution (Thermo Fisher Scientific, MA, USA) and images were captured using the Tanon 5200 Multi imaging system (Tanon, Shanghai, China).

### Immunocytochemistry (IHC)

IHC was performed as described previously [21]. In brief, MM xenograft tumors were fixed in 4% formalin, followed by paraffin embedding and serial sectioning. About 5 μm in thickness paraffin-embedded section was then dewaxed, rehydrated, and subjected to epitope retrieval. After blocking, slides were incubated with specific antibody at 4 °C overnight, followed by the incubation with secondary antibody. The results were visualized and acquired using an Olympus microscope.

### Multiple myeloma xenograft study

MM xenograft model was established with human MM cell line RPMI-8226, as described previously [22]. 3 × 10^7^ MM cells were injected subcutaneously in the flanks of each 4–6 weeks old nude mouse (Shanghai Slac Laboratory Animal Co. Ltd.). The mice were randomly divided into control group and drug treated group (30 mg/kg Glaucocalyxin A body weight) daily. The tumor volume and body weight were measured every two days. The mice were sacrificed by cervical dislocation at the end of experiment. After the mice were killed by cervical dislocation, tumor tissues were removed immediately, and tumors were excised, imaged, weighed, and then conserved for further characterization. The tumor volume = (length × width × width) × 50%. This xenograft study was approved by the Review Board on Experimental Animals of Soochow University.

### Statistical analysis

Statistical significance of differences between drug-treated and control groups was determined using the Student’s t test. The minimal level of significance was *P* < 0.05.

## Results

### GLA suppresses the proliferation of the multiple myeloma cells

The chemical structure of GLA is shown in Fig. 1A. To detect the effect of GLA on the viability of MM cells, we first analyzed the cell viability of MM cell line RPMI-8226 after treated with different concentrations of GLA at 24, 48 and 72 h. And the results revealed GLA could inhibit the growth of RPMI-8226 cell significantly (Fig. 1B). In order to detect whether GLA has effect on other MM cell lines, we also chose another four human MM cell lines (LP1, U266, OCI-MY5, and OPM2) and treated them with the indicated dose of GLA followed by MTT assay at 24, 48 and 72 h. MM also exhibited a significant dose- and time-dependently decrease in cell viability (Fig. 1C). These findings suggested that GLA displays an inhibitory effect on the proliferation of MM cells.

### GLA induces apoptosis in human multiple myeloma cells

**Fig. 2:**
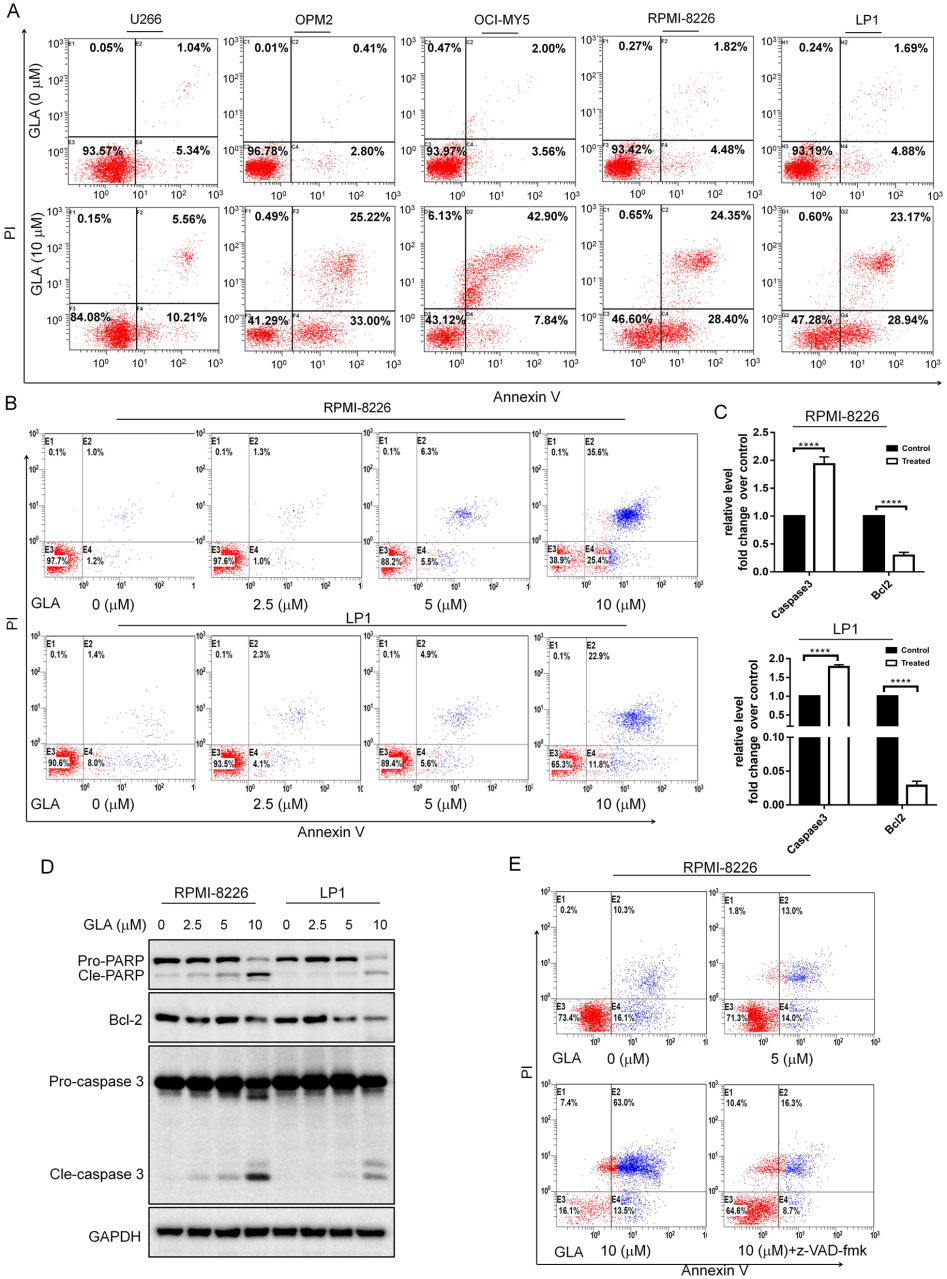
GLA induces MM cell apoptosis. **A** The six MM cell lines (U266, KMS11, OPM2, OCI-MY5, RPMI-8226 and LP1) were treated with GLA (10 μM) for 24 h, and cells were then stained with Annexin V-FITC and PI and analyzed on a BD FACSCalibur™ flow cytometer. **B** MM cell lines (RPMI-8226 and LP1) were treated with the indicated concentrations of GLA for 24 h, and cells were then stained with Annexin V-FITC and PI and analyzed by flow cytometer. **C** The levels of apoptosis related gene, Caspase-3 and Bcl-2 in MM cell lines (RPMI-8226 and LP1) treated with 10 μM GLA for 24 h were established by real time qPCR. **D** The levels of apoptosis related proteins PRAP, pro-caspase-3, and cleaved-caspase-3 and Bcl-2 in MM cell lines (RPMI-8226 and LP1) treated with different concentrations of GLA for 24 h were established by western blot analysis. **E** RPMI-8226 cells were pre-treated with or without Z-VAD-FMK (10 μM) for 3 h, and then added with the indicated concentrations of GLA (1 μM) for 24 h followed by Annexin V/PI apoptosis assay

To investigate the effect of GLA on MM cell apoptosis. The five MM cell lines (U266, OCI-MY5, OPM2, RPMI-8226 and LP1) were treated with or without GLA for 24 h, and then used to examine the apoptotic effect of GLA by Annexin V-FITC staining assay. First, the apoptotic percentage of MM cells all were more than 15% after 24 h of treatment with 10 μM GLA, especially in the OPM2 and RPMI-8226 cell line (more than 50%) (Fig. 2A). Meanwhile, GLA induced the significant increase in the apoptosis rates of RPMI-8226 and LP1 cells in a dose-dependent manner when compared with control group (Fig. 2B). Moreover, there were distinct increase in pro-apoptosis-related proteins in the highest concentration of GLA (cleaved PARP and cleaved caspase-3) and decrease in anti-apoptosis-related protein (Bcl-2) in RPMI-8226 and LP1 cells in a dose-dependent manner after treatment with GLA for 24 h (Fig. 2D). We also detected the caspase 3 and Bcl-2 mRNA expression under the treatment of GLA using qPCR. And the results also showed GLA significantly increases the caspase 3 mRNA and decreases the Bcl-2 mRNA expression (Fig. 2C).

Furthermore, Annexin V/PI staining assay showed that GLA-induced apoptosis was abolished by the addition of pan-caspase inhibitor Z-VAD-FMK in RPMI-8226 cells (Fig. 2E). All these data suggested that GLA induces the MM cell apoptosis significantly.

### GLA inhibits STAT3 activation and phosphorylation in human multiple myeloma cells

**Fig. 3:**
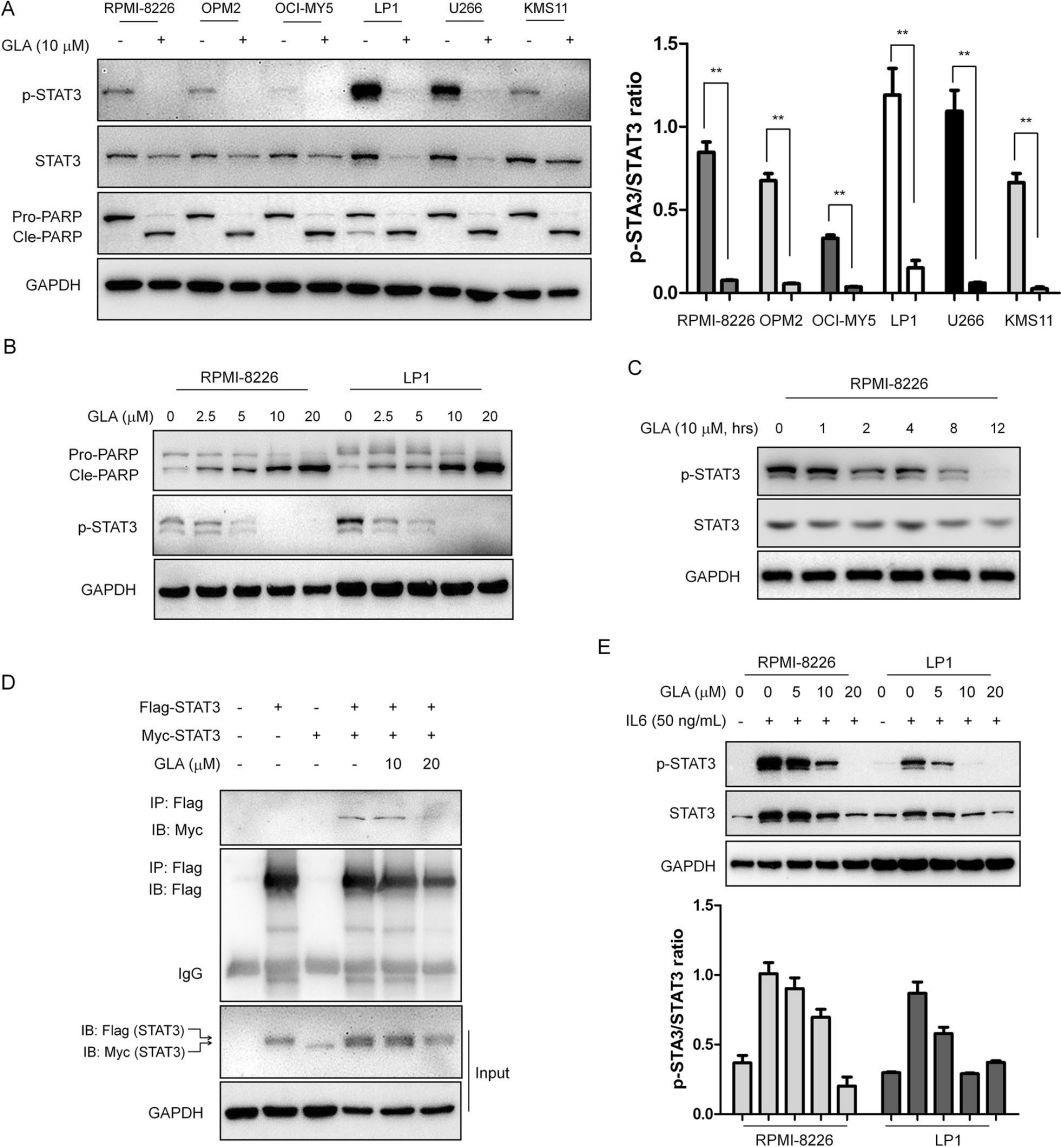
GLA inhibits STAT3 activation in human MM cells. **A** The six MM cell lines (RPMI-8226, OPM2, OCI-MY5, LP1, U266 and KMS11) were treated with GLA (10 μM) for 24 h, and the protein levels of p-STAT3, total STAT3 and PARP were detected by western blot assays. **B** RPMI-8226 and LP1 cells were treated with the indicated concentrations of GLA for 24 h, and the protein levels of p-STAT3 and total STAT3 were detected by western blot assays. **C** RPMI-8226 cells were treated with GLA at various time, and the protein levels of p-STAT3 and total STAT3 were detected by western blot assays. **D** HEK293T cells were transfected with Myc-STAT3 and/or Flag-STAT3 for 40 h, followed by GLA treatment for 8 h. Cell extracts were then prepared for immunoprecipitation (IP) with an anti-Flag antibody and subsequent western blot assay for Myc. **E** RPMI-8226 and LP1 cells were treated with GLA for 24 h, and then stimulated with IL-6 (50 ng/mL) for 15 min. The protein levels of p-STAT3 and total STAT3 were measured by western blot assay

STAT3 is an important transcription factor for MM cell survival. We detected the effect of GLA on the activation of STAT3 in MM cells. As shown in Fig. 3A, Western blot analysis showed that GLA significantly decreased the levels of p-STAT3 (Tyr705) expression without changing the levels of total STAT3 expression in MM cell lines (U266, OCI-MY5, OPM2, RPMI-8226, KMS-11 and LP1) after 24 h of treatment. GLA also induced a significant cleavage of PARP in MM cells (Fig. 3A). Moreover, to confirm the inhibition of GLA on MM cells, we treated with RPMI-8226 and LP1 cell line with different concentrations of GLA, and the results showed GLA inhibited STAT3 phosphorylation in a dose-dependent manner (Fig. 3B). And the results revealed STAT3 phosphorylation was completely suppressed by GLA treatment at 10 μM for 12 h in RPMI-8226 cells (Fig. 3C). Because the activation of STAT3 could result in STAT3 dimerization, we used Myc and Flag-tagged STAT3 plasmids to test the effect of GLA on STAT3 dimerization, and then HEK293T cells were co-transfected with these two plasmids followed by GLA treatment. Western blot assay showed that dimerization of STAT3 was observed in the control group (without high dose GLA), but this dimerization was obviously reduced after 20 μM GLA treatment for 8 h (Fig. 3D). The results implied that GLA suppressed STAT3 activation. The cytokine IL-6 can induce STAT3 activation. As shown in Fig. 3E, we observed that GLA significantly inhibited the STAT3 phosphorylation induced by the addition of IL-6 in a concentration-dependent manner in RPMI-8226 and LP1 cells. Taken together, the results suggested GLA induces the apoptosis of MM cell lines through inhibiting the phosphorylation of STAT3.

### GLA activates apoptotic signaling pathway by inhibiting STAT3 activation

**Fig. 4:**
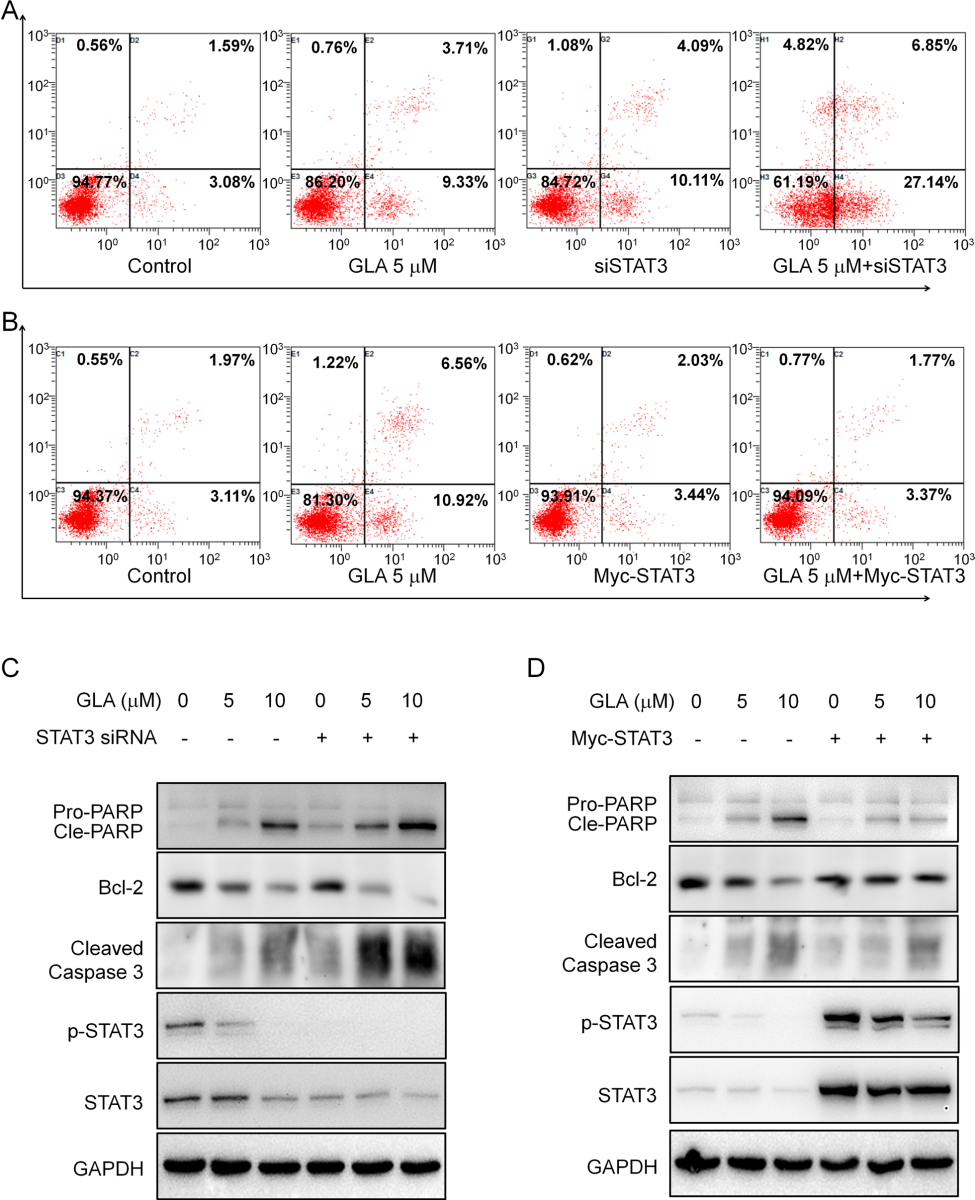
GLA activates apoptotic signaling pathway through STAT3 pathway. **A** RPMI-8226 cells were transfected with a STAT3 siRNA for 24 h followed by GLA treatment for another 24 h and then the cells were analyzed by flow cytometer. **B** RPMI-8226 cells were transfected with a STAT3 plasmid for 24 h, followed by GLA treatment for another 24 h and cells were then stained with Annexin V-FITC and PI and analyzed by flow cytometer. **C** RPMI-8226 cells were transfected with a STAT3 siRNA for 24 h, followed by GLA treatment for another 24 h. Cells were then subject to western blot assay. **D** RPMI-8226 cells were transfected with a STAT3 plasmid for 24 h followed by GLA treatment for another 24 h. Cells were then applied for western blot assay

To further confirm the effect of STAT3 in MM cell death induced by GLA, RPMI-8226 cells were transfected a STAT3 siRNA and a STAT3 plasmid for 24 h, receptively, and then followed by treatment with GLA for another 24 h. Annexin V/PI staining assay showed that STAT3 knockdown could slightly induce cell apoptosis (14.2%) and significantly aggravate GLA-induced apoptosis (33.99%) compared to control group (4.67%) (Fig. 4A). While overexpression of STAT3 markedly attenuated GLA-induced apoptosis (5.14%) compared with only GLA treated group (17.48%) (Fig. 4B). Meanwhile, we found that PARP cleavage could be slightly induced by STAT3 knockdown in the absence of GLA (Fig. 4C). Moreover, knockdown of STAT3 also significantly increased GLA-induced cleavage of PARP and Caspase 3, and meanwhile, enhanced the inhibition of Bcl-2 by GLA (Fig. 4C), while overexpression of STAT3 suppressed GLA-induced cleavage of PARP and Caspase 3, and Bcl-2 inhibition (Fig. 4D). These results showed that STAT3 participates in the MM cell death induced by GLA.

### GLA induces cell cycle arrest in human multiple myeloma cells

**Fig. 5:**
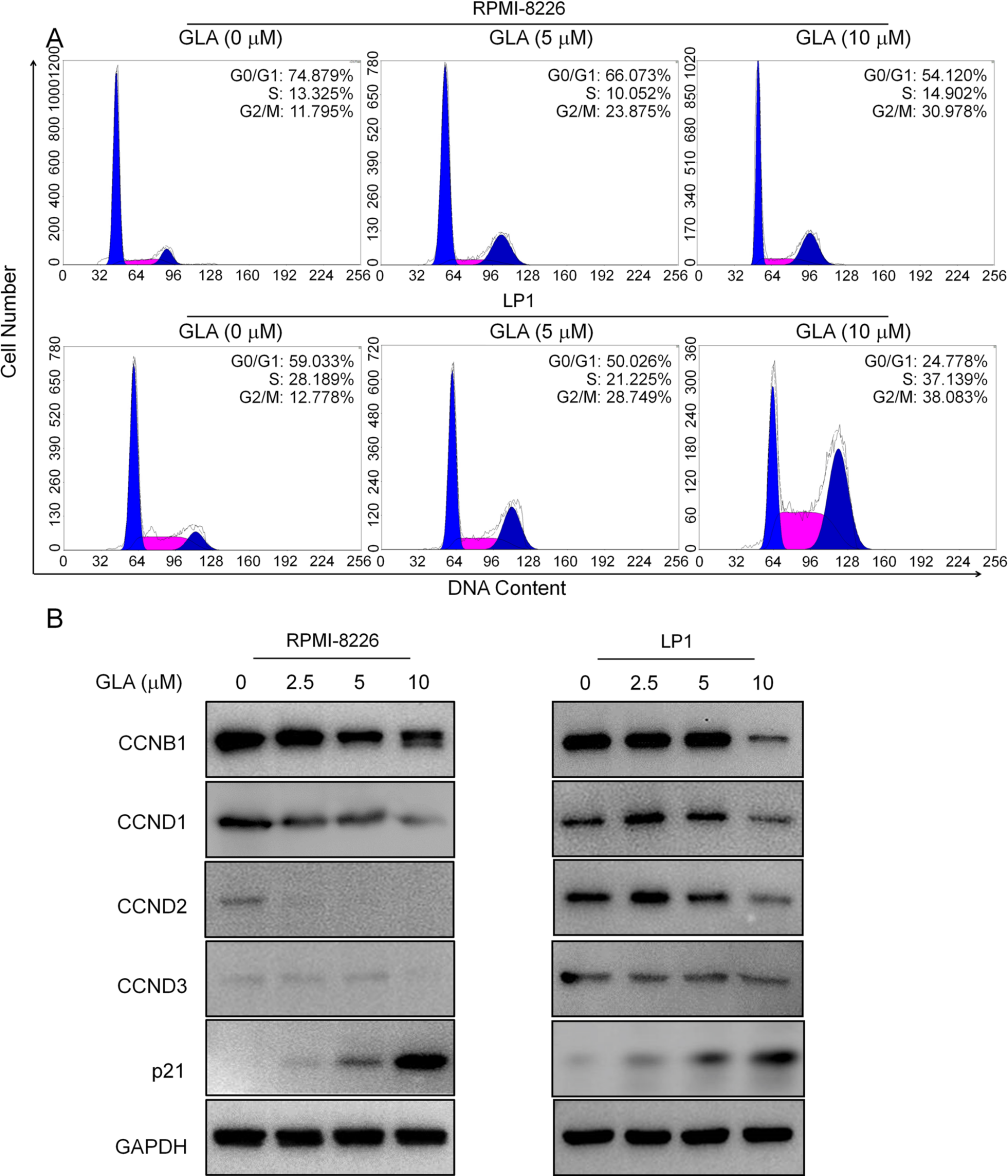
GLA induces MM cell cycle G2/M arrest. **A** MM cell lines (RPMI-8226 and LP1) were treated with various indicated concentrations of GLA for 12 h. And the cells were then stained with PI and analyzed by flow cytometer. **B** The cell cycle-related proteins CCNB1, CCND1, CCND2 and CCND3 in MM cell lines (RPMI-8226 and LP1) were measured by western blot assay

It has previously been reported that STAT3-regulated cell cycle gene are involved in myeloma cell growth [23]. We first detected the effect of GLA on MM cell cycle. RPMI-8226 and LP1 cells were treated with different concentrations of GLA for 24 h, and then evaluated by flow cytometry and Western blot assay. We observed that GLA induced a dose-dependent decrease in the percentage of G0/G1 phase cells and a dose-dependent increase in the percentage of G2/M phase cells compared with the control group in RPMI-8226 and LP1 cells (Fig. 5A). Meanwhile, Western blot assay showed that GLA significantly decreased the expression of cell cycle-related proteins CCNB1, CCND1, CCND2 and CCND3 and increased the expression of p21 compared with the control group in RPMI-8226 and LP1 cells (Fig. 5B). These results suggested that GLA induces MM cell-cycle arrest in G2/M phase.

### GLA inhibits myeloma tumor growth in a xenograft model

**Fig. 6:**
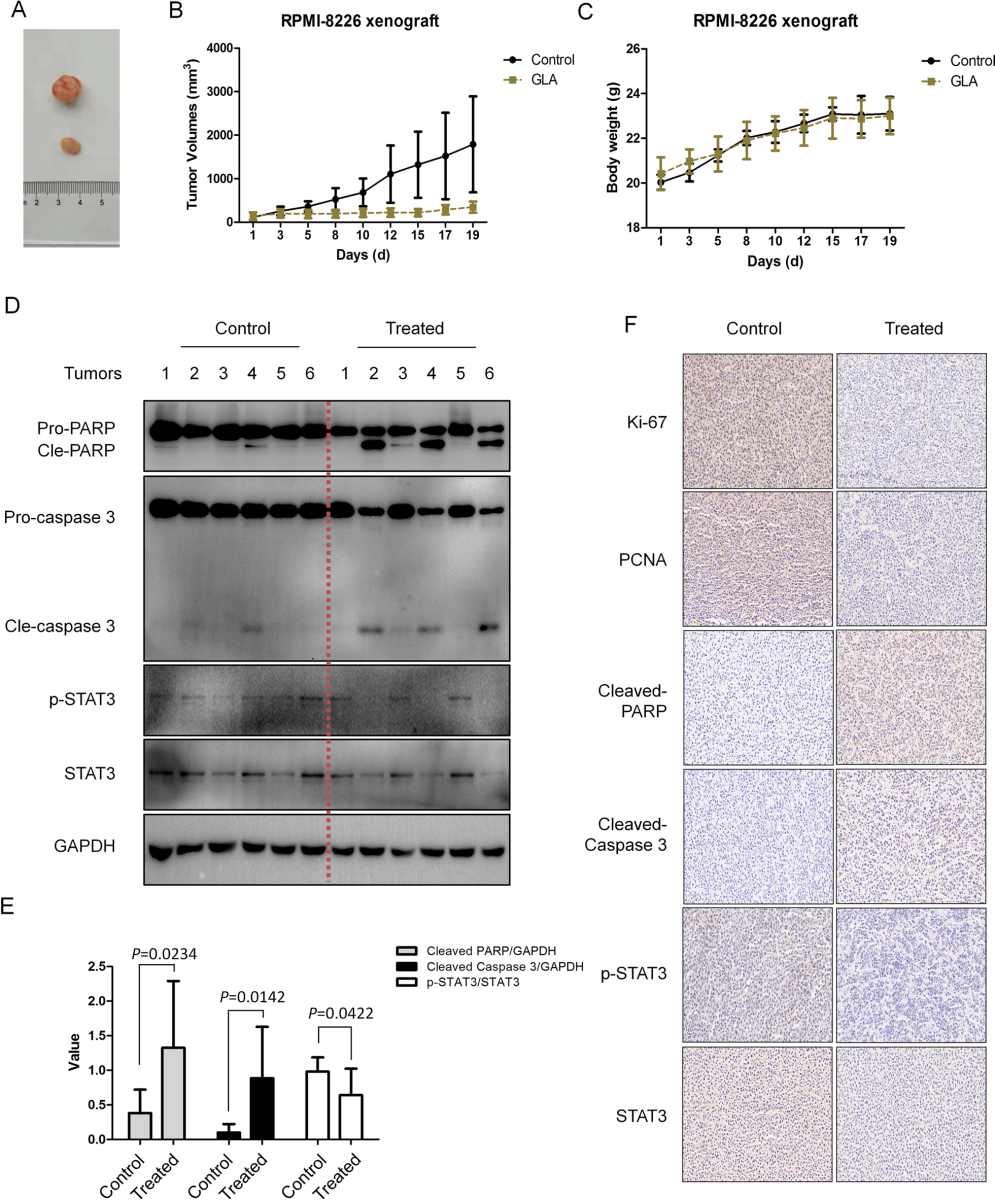
GLA suppresses myeloma tumor growth in xenograft mice models. **A** Human MM cells RPMI-8226 were injected subcutaneously into nude mice with a density of 3 × 10^7^ cells/site/mouse. When tumors were palpable, mice (n = 6/group) were intraperitoneally given GLA (30 mg/kg body weight) in PBS containing 10% Tween 80 and 10% DMSO daily for continuous 19 days. The image of nude mice xenograft tumors was captured after the treatment of GLA for 19 days. Tumor volumes (**B**) and mouse body weight (**C**) were monitored every two days. Data are shown as the means ± SD. **D** The tumor tissues extracted were prepared for western blot assay. The protein level of PARP, pro-Caspase-3, cleaved-Caspase-3, p-STAT3 and STAT3 were measured. **E** Quantitative analysis of the expression of cleaved PARP, cleaved Caspase 3, p-STAT3 and STAT3. **F** IHC analysis was used to visualize tumor proliferation maker Ki-67, PCNA, and apoptosis related genes cleaved PARP, cleaved Caspase 3, and STAT3 pathway key genes p-STAT3, STAT3. Scale bar, 20 μm

To further investigate the anti-myeloma efficacy of GLA in vivo, we established a xenograft mouse model by subcutaneous injection of RPMI-8226 cells into the nude mice. Nude mice were administered by intraperitoneal injection of GLA (30 mg/kg) or vehicle daily for about 3 weeks. Tumor volumes of mice were monitored by every other day. GLA significantly suppressed the tumor growth compared with the control group (Fig. 6A and B). However, body weight in both GLA-treated mice group and control mice group have no marked differences during the whole experiment (Fig. 6C). Moreover, the Western blot results showed GLA significantly reduced p-STAT3 expression and increased cleaved PARP and cleaved caspase-3 in tumor tissues compared with the control group (Fig. 6D). In Fig. 6E using gray quantitative analysis of the expression of cleaved PARP, cleaved Caspase 3 compared to control, and p-STAT3/STAT3 ratio, and the results confirmed that GLA significantly inhibited the phosphorylation of STAT3 and induced apoptosis in MM xenograft model. Furthermore, using IHC method, the cell proliferation markers Ki-67, PCNA and the key protein of STAT3 pathway p-STAT3 was significantly decreased, the apoptosis related gene cleaved Caspase 3 and PARP were significantly increased by GLA (Fig. 6F), further suggesting that GLA inhibited the cell proliferation in multiple myeloma through STAT3 pathway. All these data implied that GLA significantly delays multiple myeloma tumor growth in vivo xenograft model.

## Discussion

In our study, we found the ent-kauranoid diterpene derived from *Rabdosia japonica var,* Glaucocalyxin A (GLA), displays potent inhibition role against MM through inhibiting the STAT3 signal pathway in vitro and in vivo.

Apoptosis, necrosis and autophagy are believed as the main three types of cell death and play a vital role in a variety of cancers [24]. Many anti-cancer drugs, including many Chinese herb extracts, inhibit the growth of cancer cells through inducing cancer cells apoptosis, necrosis, autophagy. In our study, we first used MTT method and found GLA inhibited the growth of MM cell lines, including RPMI-8226, U266, LP1, OCI-MY5 and OPM2. And we also detected the apoptosis in GLA-treated MM cells. In the Annexin-V/PI staining assay, GLA induced significant apoptosis in different multiple myeloma cell lines (Fig. 2A) and GLA-treated cells also showed a dose-dependent increase of apoptosis in RPMI-8226 and LP1 cells (Fig. 2B). And addition of a pan-caspase inhibitor (Z-VAD-FMK) could attenuate GLA-induced MM cell apoptosis in MM cell line RPMI-8226 (Fig. 2E). Furthermore, we also found that GLA increased apoptosis related genes Caspase-3 and PARP protein and mRNA (Fig. 2C) and decreased the mRNA and protein expression of Bcl-2 (Fig. 2D).

There are many important signal pathways in the process of multiple myeloma, such as STAT3 pathway. STAT3 signal pathway activation contains several key steps. Upon stimulation, such as growth factors and cytokines, STAT3 is often phosphorylated and then forms a homo- or hetero-dimer. And then the dimers subsequently translocate to the nucleus, and then they binds to their target genes and activates or inhibits their transcription, such as *Bcl-2* [25]. So based on this, many drugs, such as HO-3867, were designed and showed inhibition of the function of STAT3 through blocking STAT3 phosphorylation, transcription or DNA binding activity [26]. In our study, we used Western blot and Immunoprecipitation methods and found, GLA significantly inhibited the STAT3 phosphorylation in different MM cell lines (Fig. 3A), meanwhile the inhibition was dose- and time-dependent with or without stimulation by IL6 (Fig. 3B, C and E). GLA could interrupt the Myc-STAT3:Flag-STAT3 interaction, which suggested this inhibition by GLA maybe due to the disruption of STAT3 dimerization (Fig. 3D). Moreover, knocking out or overexpression of STAT3 can enhance or abolish the apoptosis of MM cell lines induced by GLA using Annexin V-FITC assay and Western blot methods, which further suggested STAT3 was the target of GLA (Fig. 4).

As a critical transcription factor, STAT3 modulates a broad panel of important genes transcription and expression, such as *Bcl-2, Mcl-1*, *survivin* and *XIAP* (that are apoptosis related genes), cyclin-dependent kinase, transcription factor *E2F-1* (that are cell cycle related regulators) and D-type cyclins (that are universally dysregulated in MM cells) [8, 27–30]. It is well known that the cell cycle consists of four phases, the G0/G1, S, G2/M and M phases. Various anticancer drugs, such as cisplatin and paclitaxel, have been recently applied to induce cancer cell death by interfering with cell cycle checkpoints, especially G2/M checkpoints [31, 32]. To examine the role of GLA on MM cell cycle, we detected the MM cell cycle under stimulation by GLA using flow cytometry. Our study revealed that GLA promoted the G2/M phase arrest via regulating the G2/M checkpoints, including downregulation of cyclin B1, CCND2, CCND1, and upregulation of P21 in vitro (Fig. 5A and B). GLA thus decreased proliferation and increased apoptosis of MM cells through blocking the STAT3 pathway.

Finally, to investigate the anticancer effect of GLA in vivo, we constructed the xenograft mouse model, and our results revealed that GLA can significantly inhibit the growth of multiple myeloma (Fig. 6B). And the mice xenograft tissues also showed GLA significantly inhibited the expression of STAT3, which is consistent with the results in vitro (Fig. 6D and E). The IHC results also confirmed that GLA inhibited the cell proliferation marker Ki67 and PCNA. And the expression of p-STAT3 was significantly downregulated in the treated group compared to the control group in vivo (Fig. 6F). In addition, no obvious body weight change was observed in the GLA-treated MM mice model (Fig. 6C), which suggests the low toxicity of GLA on MM xenograft mice model.

**Fig. 7:**
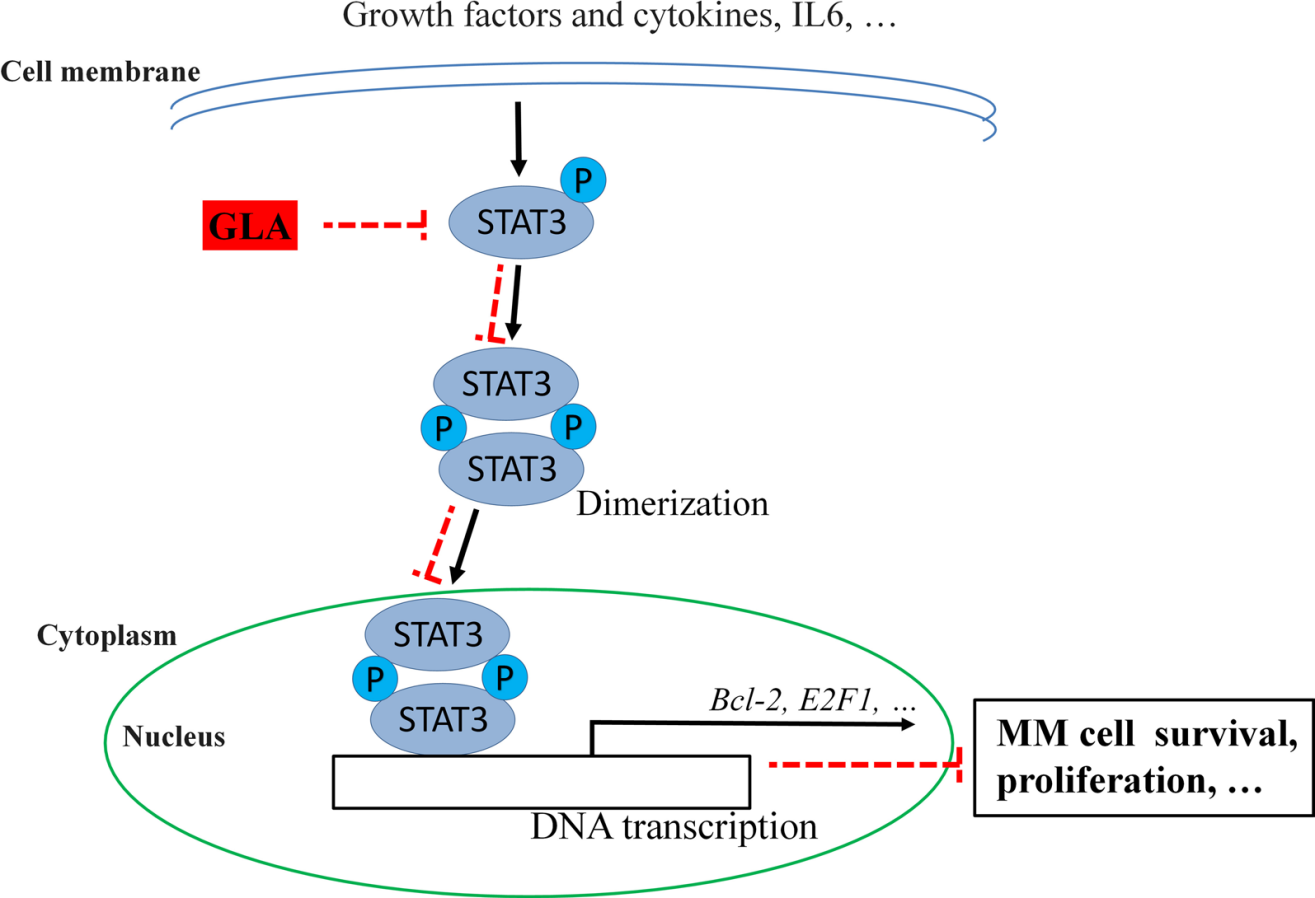
Schematic summary of probably molecular mechanisms of inhibition by GLA in MM

In conclusion, as shown in Fig. 7, our findings showed that GLA can significantly suppress the growth of MM through blocking phosphorylation of STAT3 and then inducing the apoptosis of MM cell line in vitro and in vivo. Our findings for the first time clearly demonstrate that GLA may be developed as a potential anti-cancer drug for the treatment of MM in clinical in the future.

## Data Availability

All data are included in the article.

## Abbreviations

Bcl2: B-Cell CLL/lymphoma 2
CCNB1: Cyclin B1
CCND1: Cyclin D1
CCND2: Cyclin D2
CCND3: Cyclin D3
Mcl-1: Myeloid cell leukemia 1
FBS: Fetal bovine serum
GLA: Glaucocalyxin A
MM: Multiple myeloma
MTT: 3-(4:5-Dimethylthiazol-2-yl)-2:5-diphenyltetrazolium bromide
PAGE: SDS–polyacrylamide gel electrophoresis
STAT3: Signal transducer and activator of transcription 3
